# Abscess of the Brain

**Published:** 1901-06-01

**Authors:** 


### ABSCESS OF THE BRAIN.

By far the most common source of cerebral abscess is suppuration within the middle ear. It was then only appropriate that in addressing the last meeting of the Otological Society Mr. Charles A. Balance should choose for his subject the operative treatment of abscess when situated in the brain. Eliminating from his subject all taint of specialism Mr. Balance held with Stäcke that "otology is an offshoot of surgery, and only in close adherence to it, and in the true and conscientious observance of its principles is success to be sought for and to be found," and with Da Costa that "in the treatment of an abscess there is one absolute rule which knows of no exception—viz., that whenever and wherever pus is found the abscess should be evacuated at once, and after evacuating it thorough drainage must be provided for." To these maxims, however, Mr. Balance adds that when a purulent collection is encapsuled enucleation is the proper treatment. Coming to the details of the operation required in cases of abscess of the brain, and taking for granted that the local source of infection has already been thoroughly removed, he describes the steps of the operation as including the following:—

1. *Sterilisation of the Skin.*

2. *Anæsthesia.*—The anæsthetic should be chloroform, and it should be given warily, for in cerebral abscess the respiration is apt to cease suddenly.

3. *Incision of the Scalp.*—A flap is better than a crucial incision. It should have its base downwards, and should be considerably larger than the bony opening.

4. *Opening in the Bone.*—This should be a free one, and should be so planned as to allow free drainage downwards. After the skull has been opened by the trephine additional bone may have to be removed by saws, forceps, or Cryer's drill.

5. *Incision of the Dura Mater.*—A flap is preferable to a crucial incision, and its base should be upwards.

6. *Discovery of the Abscess.*—If by palpation it can be ascertained that the abscess is immediately subcortical, an incision should be at once made without the preliminary use of any pus-seeking apparatus, such as trocar and canula, or the like; while if the seat of the abscess be not obvious it must be sought for, the best instrument for this purpose being a sharp-pointed, long and narrow knife. If careful exploratory puncture fail to find the abscess, the finger, inserted into the brain substance will almost infallibly detect the presence of a tense abnormal swelling.

7. *The Further Treatment of the Abscess.*—This includes the details in regard to drainage, which must be efficient. In using gauze care must be taken not to plug the orifice as well as the cavity, and if the abscess has been so deep a one as to have led to the use of a trocar and canula the canula must not be withdrawn, but must remain as a drain. "Many a case has been lost after the pus has been evacuated owing to failure to reintroduce the tube in the proper position."

8. *Closure of the Wound.*—An aperture is made in the base of the flap the size of the opening in the brain, and the edge of the flap replaced in position and sutured with silkworm gut. A dry sterilised cyanide dressing is recommended.

